# DNMT1 regulates expression of MHC class I in post-mitotic neurons

**DOI:** 10.1186/s13041-018-0380-9

**Published:** 2018-07-03

**Authors:** Julie Ry Gustafsson, Georgia Katsioudi, Matilda Degn, Patrick Ejlerskov, Shohreh Issazadeh-Navikas, Birgitte Rahbek Kornum

**Affiliations:** 1 Department of Clinical Biochemistry, Molecular Sleep Laboratory, Rigshospitalet, Glostrup, Nordre Ringvej 57, 2600 Glostrup, Denmark; 2 Biotech Research and Innovation Centre (BRIC), University of Copenhagen, Ole Maaløes Vej 5, 2200 Copenhagen N, Denmark; 3 Department of Clinical Neurophysiology, Danish Center for Sleep Medicine, Rigshospitalet, Glostrup, Denmark; 4 Molecular Sleep Laboratory, Faculty of Health and Medical Sciences, University of Copenhagen, Copenhagen, Denmark

**Keywords:** MHC class I, Post mitotic neurons, DNMT1, HLA, H2, Autoimmune neurodegeneration

## Abstract

Major Histocompability Complex I (MHC-I) molecules present cellularly derived peptides to the adaptive immune system. Generally MHC-I is not expressed on healthy post-mitotic neurons in the central nervous system, but it is known to increase upon immune activation such as viral infections and also during neurodegenerative processes. MHC-I expression is known to be regulated by the DNA methyltransferase DNMT1 in non-neuronal cells. Interestingly DNMT1 expression is high in neurons despite these being non-dividing. This suggests a role for DNMT1 in neurons beyond the classical re-methylation of DNA after cell division. We thus investigated whether DNMT1 regulates MHC-I in post-mitotic neurons. For this we used primary cultures of mouse cerebellar granule neurons (CGNs). Our results showed that knockdown of *DNMT1* in CGNs caused upregulation of some, but not all subtypes of MHC-I genes. This effect was synergistically enhanced by subsequent IFNγ treatment. Overall MHC-I protein level was not affected by knockdown of *DNMT1* in CGNs. Instead our results show that the relative MHC-I expression levels among the different MHC subtypes is regulated by DNMT1 activity. In conclusion, we show that while the mouse *H2-D1/L* alleles are suppressed in neurons by DNMT1 activity under normal circumstances, the *H2-K1* allele is not. This finding is particularly important in two instances. One: in the context of CNS autoimmunity with epitope presentation by specific MHC-I subtypes where this allele specific regulation might become important; and two: in amyotropic lateral sclerosis (ALS) where *H2-K* but not *H2-D* protects motor neurons from ALS astrocyte-induced toxicity in a mouse model of ALS.

**Electronic supplementary material:**

The online version of this article (10.1186/s13041-018-0380-9) contains supplementary material, which is available to authorized users.

## Introduction

Involvement of the immune system is a common trait in several neurodegenerative diseases and autoimmune diseases which affect the central nervous system (CNS) [1, 2], and the etiology of these neurological disorders is often not determined. Many autoimmune diseases are associated with certain Major Histocompability Complex (MHC) alleles, including type 1 narcolepsy that has a very strong association with HLA-DQB1*06:02 and are also associated with certain HLA class I alleles [3, 4]. MHC class I signaling has also been suggested to play a role in neurodegenerative disorders such as Parkinson’s disease and Amyotrophic Lateral Sclerosis (ALS) [5, 6]. Our goal with the current study was to thus study the regulation of MHC class I (MHC-I) in neurons.

The CNS was originally considered an immune privileged organ [7], however this perception is no longer prevalent. We now know that peripheral T cells can enter the CNS, both when recruited by chemokines upon viral infection of neurons [8] and also as routine monitoring where memory T cells can be reactivated by antigen-presenting dendritic cells in the perivascular and subarachnoid space [9]. A range of autoimmune CNS diseases involving neuronal destruction is likely mediated by CD8^+^ T cells, as suggested by the presence of CD8^+^ T cells in affected brain areas in Rasmussen’s encephalitis [10], paraneoplastic neuronal degeneration [11], and Multiple Sclerosis [12] which is otherwise considered a CD4^+^ T cell driven disease. CD8^+^ T cell mediated neuronal destruction depends however on activation by interaction with MHC-I molecules, which are not expressed on healthy post-mitotic neurons under non-pathological conditions [13, 14]. MHC-I is in contrast expressed in neurons at early developmental stages but gets downregulated in adulthood [15–17]. During development, MHC-I molecules have been shown to negatively regulate synaptic density [18], and the establishment of cortical connections [19]. In adulthood, the same functions seem to play a role under different pathological conditions. In the middle cerebral artery occlusion (MCAO) model of stroke, MHC-I knock out mice (*H2-Kb* and *H2-Db* double knock out) have smaller infarct areas and better behavioral recovery [20]. In contrast, under conditions of axonal injury, MHC class I molecules seem to play a beneficial role and have for instance been found to protect specific synaptic contacts from detachment [21].

MHC-I molecules are readily induced on neurons upon interferon gamma (IFNγ) treatment [13, 14], and is also regulated by neuronal activity during development [16, 22, 23]. Several studies have shown that when MHC-I is induced on neurons, CD8^+^ T cells can recognize and kill the MHC-I-expressing neurons subsequently [24–26]. MHC-I molecules have also been shown to play a role in neurodegenerative disease. A common theme here is that MHC-I molecules are higher on vulnerable neuronal cell types, such as dopaminergic neurons (Parkinsons disease) and motor neurons (ALS). In these cases MHC-I expression seems to be protective and is downregulated with disease progression [5, 6]. Thus, insight into the mechanisms keeping MHC-I expression on neurons in check is of interests for a variety of CNS diseases.

A missense mutation in the DNA methyltransferase 1 gene (*DNMT1*) causes the neurodegenerative disease ADCA-DN (Autosomal dominant cerebellar ataxia, deafness and narcolepsy), where three neuronal cell types particularly vulnerable to autoimmunity are lost: the hypocretin neurons (lost in narcolepsy), the cerebellar Purkinje neurons (lost in paraneoplastic cerebellar neurodegeneration), and neurons of the inner ear (lost in autoimmune hearing loss) [27], showing that changes in DNMT1 activity affects post mitotic neurons with devastating consequences.

Our specific aim was thus to investigate whether DNMT1 activity regulates MHC-I expression on neurons. We here provide proof-of-principle data using neuroblastoma cell lines and next show that the mechanism also exists in post mitotic neurons. We chose cerebellar granule neurons (CGNs) as a model, as the neurons are prepared from postnatal pups and the neurons in culture are post-mitotic [28, 29]. *DNMT1* mRNA have been detected at high levels at this stage in cerebellum [30], and DNMT1 protein have been detected in CGNs from mice [31].

## Results

### Inhibitors of DNMT1 increase MHC-I gene expression in dividing human and mouse neuroblastoma cell lines

**Fig. 1:**
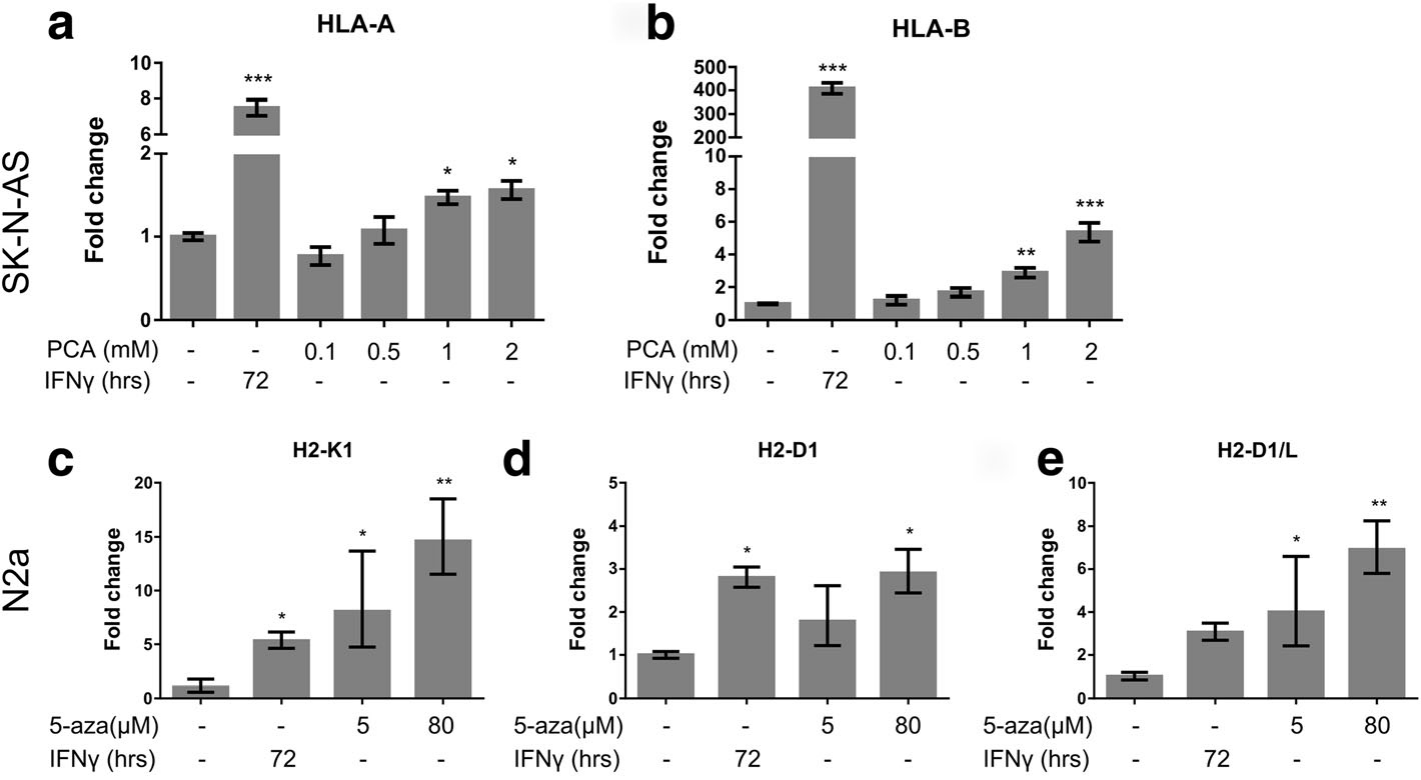
Inhibitors of DNMT1 increase MHC-I gene expression in neuroblastoma cell lines. PCA dose-dependently increased *HLA-A* gene expression (**a**) and *HLA-B* gene expression (**b**) in human neuroblastoma cell line SK-N-AS. 5-aza dose-dependently increased the gene expression of *H2-K1* (**c**), *H2-D1* (**d**), and *H2-D1/L* (**e**) in mouse neuroblastoma cell line N2a. 72 h treatment with IFNγ (0,2 ng/uL in SK-N-AS and 0,5 ng/uL in N2a) significantly increases gene expression of *HLA-A* (**a**), *HLA-B* (**b**), *H2-K1* (**c**) and *H2-D1* (**d**). *H2-D1/L* gene expression was increased, but not significantly (*p* = 0.06) (**e**). Bars represent mean +/− S.E.M., *N* = 3. * *p* < 0.05, ** *p* < 0.01, *** *p* < 0.001 vs. untreated in a One-Way ANOVA with Dunnett’s post hoc test

To test whether MHC-I genes are regulated by DNMT1 in neuron-like cell lines, we added inhibitors to both human and mouse neuroblastoma cell lines and analyzed gene expression of the MHC-I genes. Procainamide (PCA) is a known inhibitor of DNMT1 [32]. Treatment with PCA for 72 h, dose-dependently induced significant *HLA-A* and *HLA-B* gene expression in human SK-N-AS (Fig. 1a and b). Gene expression of *HLA-A* was increased 1.5 fold by both 1 mM and 2 mM PCA compared to untreated cells (*p* = 0.036, and *p* = 0.014 respectively) (Fig. 1a). Gene expression of *HLA-B* was increased 2.9 fold by 1 mM PCA (*p* = 0.0083) and 5.5 fold by 2 mM PCA (*p* < 0.001) (Fig. 1b). Basal relative expression level of HLA-A was 17.1% while the basal level of HLA-B was 0.03%, compared to endogenous expression levels of β-Actin (ACTB) and GAPDH. *HLA-C* was not detectable in these cells. The basal relative levels of other relevant genes were: *β2M* = 30.7%, *DNMT1* = 5.0%, *DNMT3a* = 2.7%*,* and *DNMT3b* = 0.01%.

We also tested whether knocking down *DNMT1* or the other DNA methyltransferases *DNMT3a* and *DNMT3b,* in human SK-N-AS cells would affect expression levels of HLA-A and HLA-B. We evaluated the gene expression compared to non-targeting siRNA (baseline, Additional file 1: Figure S1A-F) for each of the genes of interests *HLA-A*, *HLA-B*, and *β2M*, as well as of the targeted genes *DNMT1*, *DNMT3a* and *DNMT3b*, upon treatment for 72 h with either non-targeting siRNA, siRNA against *DNMT1*, siRNA against *DNMT3a*, or siRNA against *DNMT3b* (Additional file 1: Figure S1). Specific downregulation of the targeted DNMT was obtained for all three *DNMTs*. *DNMT1* gene expression was decreased by 43% upon treatment with siRNA against *DNMT1* (*p* = 0.019 versus non-targeting siRNA) (Additional file 1: Figure S1A). This resulted in a significant upregulation of *HLA-A* expression by 50% (*p* = 0.0061 versus non-targeting siRNA) (Additional file 1: Figure S1D). We observed a non-significant tendency towards upregulation of *HLA-B* and *β2M* as well. *DNMT3a* levels was decreased by 32% upon treatment with siRNA against *DNMT3a* (*p* = 0.017 versus non-targeting siRNA) (Additional file 1: Figure S1B), and *DNMT3b* levels was decreased by 48% upon treatment with siRNA against *DNMT3b* (*p* = 0.029 versus non-targeting siRNA) (Additional file 1: Figure S1C). Neither of these changes resulted in significant effects on *HLA-A*, *HLA-B* or *β2M,* even though we did see tendencies towards upregulation when DNMT3b was knocked down.

To validate whether a similar regulation of MHC-I genes also existed in mouse cells, we evaluated the expression of *H2* genes in mouse N2a neuroblastoma cell lines upon 72 h of treatment with the inhibitor of DNMT1 and DNA methylation 5-aza-2-deoxycytidine (5-aza). The N2a cell line is derived from strain A mice and thus carries the HLA-I subtypes *H2-Kk*, *H2-Dd*, and *H2-Ld*. We used 5-aza rather than PCA in the N2a cell lines, as 5-aza has been used regularly in N2a neuroblastoma cell lines [33, 34], and as we experienced an unintended (and likely compensatory) increase in *DNMT3b* gene expression level upon treatment with PCA (data not shown). *H2-K1* and *H2-D1/L* gene expression levels were significantly increased by both 5 μM and 80 μM 5-aza compared to no treatment (*H2-K1* increased 8.1 fold by 5 μM (*p* = 0.018), *H2-K1* increased 14.6 fold by 80 μM (*p* = 0.0044), *H2-D1/L* increased 4.0 fold by 5 μM (*p* = 0.023), and 9.1 fold by 80 μM (*p* = 0.0037) (Fig. 1c and e). *H2-D1* was only significantly increased by 80 μM to 2.9 fold compared to untreated (*p* = 0.021) (Fig. 1d). Basal relative expression levels of the genes compared to endogenous expression levels of ACTB and GAPDH were: *β2M* = 19.0%, *H2-K1* = 15.3%, *H2-D1* = 8.3%*, H2-D1/L* = 29.5%. IFNγ was used as a positive control for *HLA/H2* induction in all experiments, and induced all *HLA* and *H2* genes significantly, except for *H2-D1/L* which was induced 3.06 fold compared to untreated N2a cells (*p* = 0.06) (Fig. 1a-e). These results demonstrate that MHC-I gene expression is regulated by DNMT1 in both the human SK-N-AS and the mouse N2a neuroblastoma cell lines.

### Knockdown of DNMT1 increase MHC-I gene expression in post-mitotic mouse CGNs

**Fig. 2:**
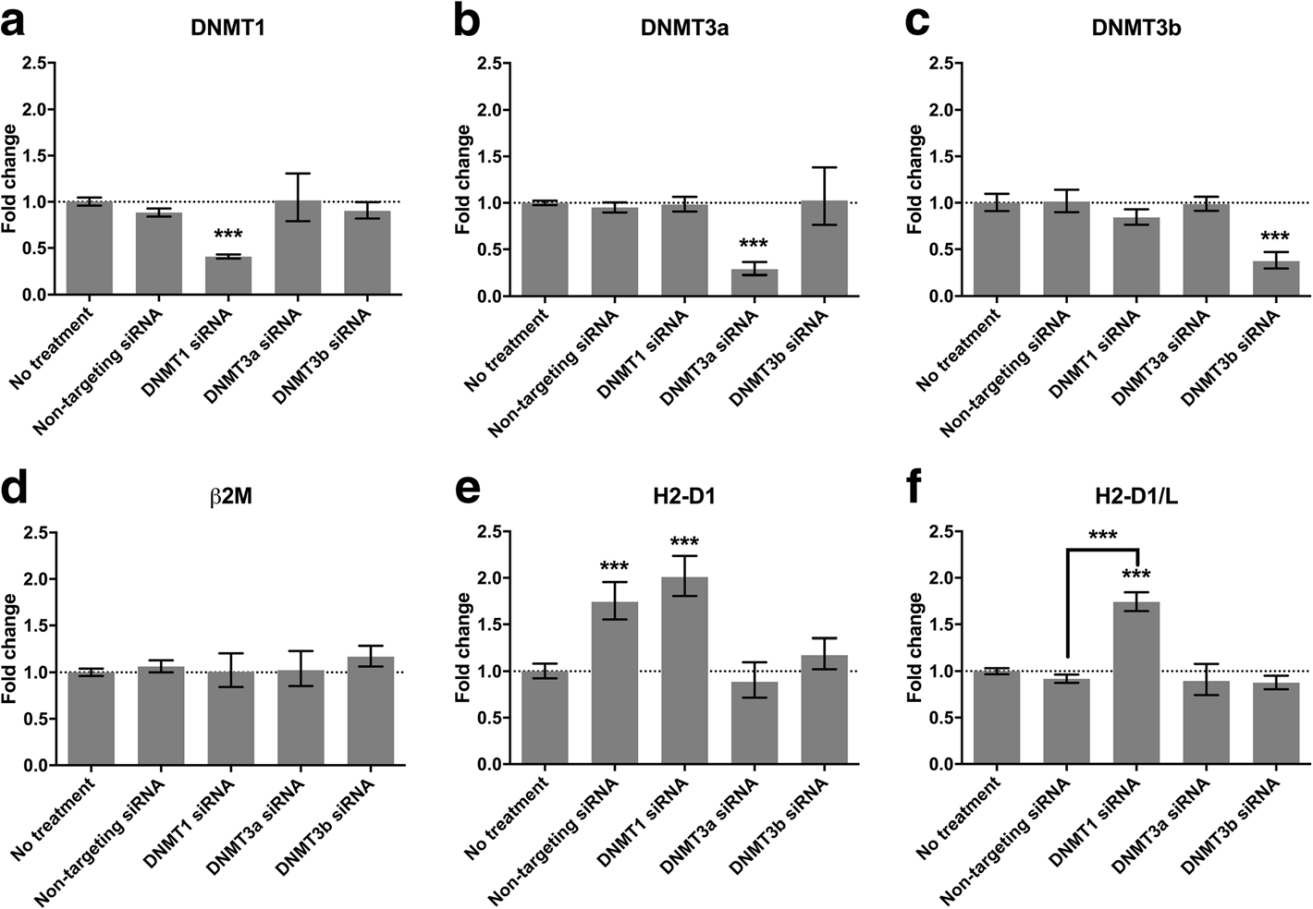
Knockdown of *DNMT1* increases MHC-I gene expression in post-mitotic mouse CGNs. Shown is the fold change in gene expression of *DNMT1* (**a**), *DNMT3a* (**b**), *DNMT3b* (**c**), *β2M* (**d**), *H2-D1* (**e**), and *H2-D1/L* (**f**) following 72 h siRNA treatment of CGN cultures. Baseline indicate gene expression level in untreated CGNs. Bars represent mean +/− S.E.M., *N* = 8 from three independent experiments. Stars illustrate effects that are significantly different from untreated CGNs, unless otherwise indicated. * *p* < 0.05, *** *p* < 0.001, multiplicity corrected *p*-values in a Two-Way ANOVA with Dunnett’s post hoc test

We next aimed at addressing whether MHC-I gene expression was also regulated by DNMT1 in post-mitotic neurons. We used CGNs for this purpose. We derived the CGNs from Balb/C mice that carry the HLA-I subtypes *H2-Kd*, *H2-Dd*, and *H2-Ld*. All known small molecule inhibitors of DNA methyltransferases are developed for dividing cells [35], and as expected PCA and 5-aza did not have any significant effect on *H2* genes in CGNs (data not shown). Thus we decided to investigate regulation of *H2* genes in CGNs by knocking down *DNMT1*, *DNMT3a* and *DNMT3b*, one at a time. We evaluated the gene expression compared to untreated CGNs (baseline 1, Fig. 2a-f) for each of the genes of interests *β2M*, *H2-K1*, *H2-D1* and *H2-D1/L*, as well as of the targeted genes *DNMT1*, *DNMT3a* and *DNMT3b*, upon treatment for 72 h with either non-targeting siRNA, siRNA against *DNMT1*, siRNA against *DNMT3a*, or siRNA against *DNMT3b* (Fig. 2a-e, bars from left to right on X-axis). In baseline conditions the relative expression levels of the different genes differed markedly in CGNs. When compared to the endogenous expression of ACTB and GAPDH, expression levels were: *β2M* = 25.5%, *H2-K1* = undetectable, *H2-D1* = 0.37%*, H2-D1/L* = 35.9%*, DNMT1* = 4.9%, *DNMT3a* = 2.6%*,* and *DNMT3b* = 0.05%.The effect of the different treatments was evaluated by statistical testing against non-targeting siRNA to exclude unspecific effects of the transfection procedure. Specific downregulation of the targeted DNMT was obtained for all three *DNMTs*. *DNMT1* gene expression was decreased by 60% upon treatment with siRNA against *DNMT1* compared to untreated (*p* < 0.0001 versus non-targeting siRNA) (Fig. 2a), *DNMT3a* levels was decreased by 71% upon treatment with siRNA against *DNMT3a* (*p* < 0.0001 versus non-targeting siRNA) (Fig. 2b), and *DNMT3b* levels was decreased by 63% upon treatment with siRNA against *DNMT3b* (*p* < 0.0001 versus non-targeting siRNA) (Fig. 2c), whereas neither *DNMT1*, *DNMT3a*, nor *DNMT3b* levels were affected significantly by other treatments (Fig. 2a-c). As observed in the neuroblastoma cell lines, the different genes encoding MHC-I responded differently to the treatments. The level of *β2M* was unaffected by any of the treatments (Fig. 2d). There was no signal from *H2-K1* in any of the samples, suggesting that this gene is not expressed in post-mitotic CGNs, and that it cannot be induced by changes in DNMT activity. Signal from the *H2-D1* probe was heavily influenced by non-targeting siRNA (Fig. 2e). Although treatment with *DNMT1* siRNA did cause a 2-fold increase in *H2-D1* gene expression compared to untreated CGNs, this effect was not significantly different from the *H2-D1* expression levels upon treatment with non-targeting siRNA (Fig. 2e). Finally, treatment with siRNA against *DNMT1* caused a 1.74 fold increase in *H2-D1/L* compared to untreated CGNs (*p* < 0.0001) (Fig. 2f), which was also significantly different from the effect of non-targeting siRNA on *H2-D1/L* (*p* < 0.0001) (Fig. 2f). Knockdown of *DNMT3a* or *DNMT3b* did not affect the expression levels of *H2-D1/L* showing that the effect is specific for *DNMT1*.

Even though Purkinje cells are notoriously difficult to culture, CGN cultures can contain a small number of Purkinje cells. We wanted to test the possibility that the observed changes was caused by large changes in the small fraction of Purkinje cells. We therefore measured the level of calbindin, a well known marker of Purkinje neurons, in our cultures. Calbindin was only borderline detectable by qPCR, and we did not observe any significant differences between the different treatments (data not shown).

### Knockdown of DNMT1 decrease markers of synaptic function in post-mitotic mouse CGNs

**Fig. 3:**
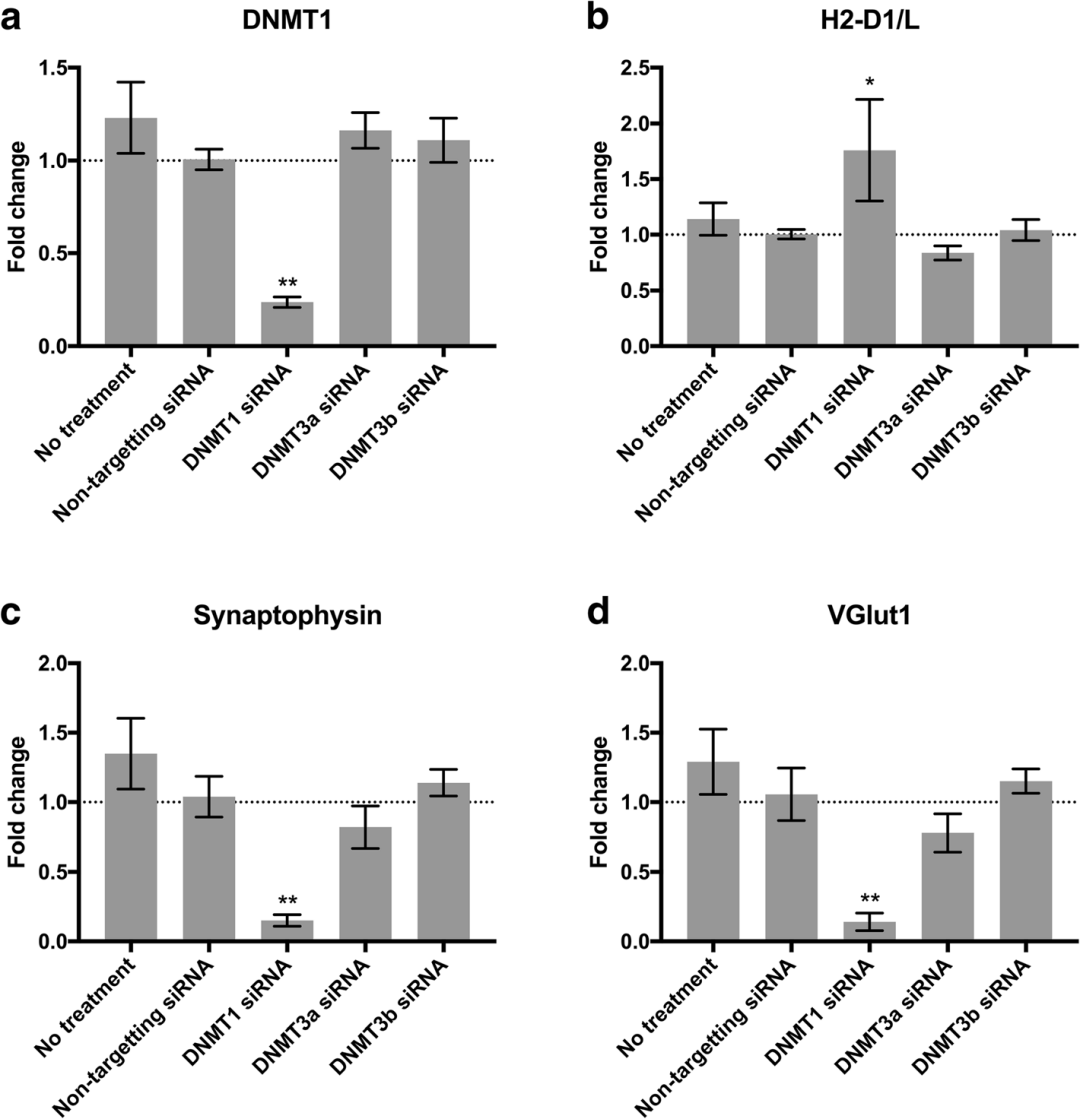
Knockdown of *DNMT1* decrease markers of synaptic function in post-mitotic mouse CGNs. Shown is the fold change in gene expression of *DNMT1* (**a**), *H2-D1/L* (**b**), synaptophysin (**c**) and VGlut1 (**d**) following 72 h siRNA treatment of CGN cultures. Baseline indicate gene expression level in CGNs treated with non-targeting siRNA. Bars represent mean +/− S.E.M., *N* = 5 from two independent experiments. Stars illustrate effects that are significantly different from non-targeting siRNA group. * *p* < 0.05, ** *p* < 0.01, multiplicity corrected p-values in a One-Way ANOVA with Dunnett’s post hoc test

Since MHC-I plays a role in synaptic plasticity during development [18, 19], and further has been suggested to also aid in synaptic pruning in the adult brain [21], we speculated whether the mechanism for upregulation of *H2-D1/L* following DNMT1 knock down, could be linked to changes in synaptic function in the cultures. To address this we measured two markers of synaptic function: synaptophysin and the vesicular glutamate transporter 1 (VGlut1). Synaptophysin is important for efficient synaptic vesicle trafficking [36] and for activity-dependent synapse formation [37], while VGlut1 is involved in glutamate vesicular release in mature CGNs [38]. In the experiment, *DNMT1* gene expression was decreased by 76% upon treatment with siRNA against *DNMT1* compared to non-targeting siRNA (*n* = 5, *p* = 0.0011) (Fig. 3a), while *H2-D1/L* was increased 1.76 fold compared to non-targeting siRNA treatment of the CGNs (*p* = 0.049) (Fig. 3b). In the same experiment, DNMT1 siRNA treatment caused a 89% downregulation of synaptophysin (*p* = 0.0022) and VGlut1 was decreased by 92% (*p* = 0.0018) (Fig. 3c and d). These results show that knock down of DNMT1 significantly affects expression of synaptic markers, while knock-down of DNMT3a or DNMT3b has no effect.

### Blockade of DNMT1 acts in synergy with IFNγ to induce MHC-I gene expression

**Fig. 4:**
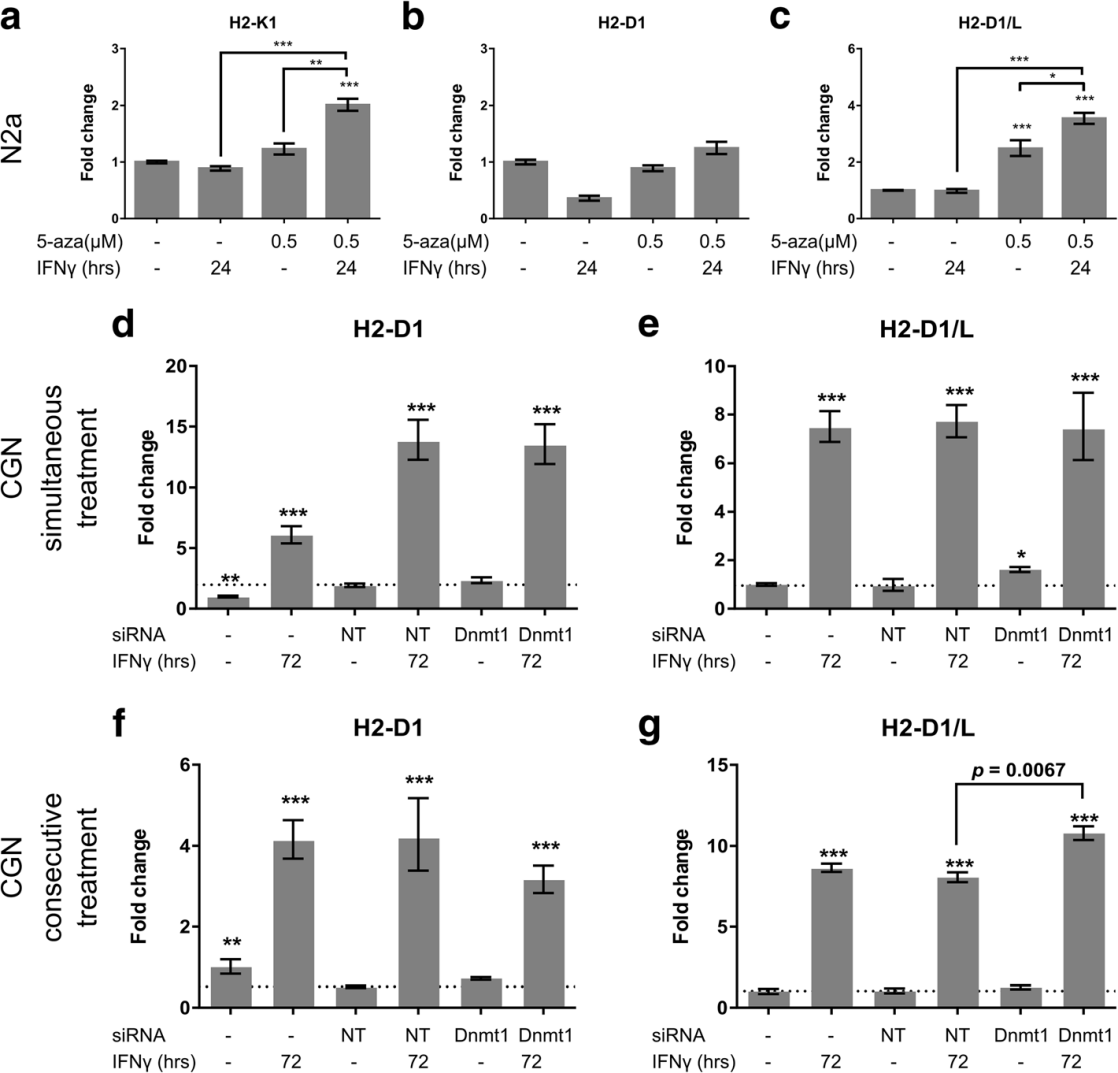
Blockade of DNMT1 acts in synergy with IFNγ to induce MHC-I gene expression. Panel A-C show the effect of combined treatment of N2a cells with IFNγ and 5-aza. Fold change in gene expression of *H2-K1* (**a**), *H2-D1* (**b**), and *H2-D1/L* (**c**) relative to untreated N2a cells. Bars represent mean +/− S.E.M., N = 3. Panel D-E show the effect of simultaneous treatment of CGNs with IFNγ and siRNA against *DNMT1* or non-targeting (NT) control siRNA. Fold change in gene expression of *H2-D1* (**d**), and *H2-D1/L* (**e**) relative to untreated CGNs. Bars represent mean +/− S.E.M., *N* = 6 from two independent experiments. Panel F-G show the effect of treatment of CGNs with siRNA against *DNMT1* for 72 h, followed by treatment with IFNγ for the next 72 h. Fold change in gene expression of *H2-D1* (**f**), and *H2-D1/L* (**g**) relative to untreated CGNs. Bars represent mean +/− S.E.M., N = 3. Baselines indicate gene expression level in CGNs treated with non-targeting (NT) siRNA. Stars in A-C illustrate effects that are significantly different from untreated CGNs, and stars in D-G illustrate effects that are significantly different from CGNs treated with non-targeting siRNA, unless otherwise indicated. * *p* < 0.05, ** *p* < 0.01, *** *p* < 0.001, multiplicity corrected p-values in a One-Way ANOVA with Dunnett’s post hoc test

IFNγ is a known inducer of MHC-I expression on neurons. It has been reported that IFNγ and the demethylating agent zebularine work in synergy to increase expression of indoleamine 2,3-dioxygenase 1 (*IDO1*) -another gene known to be induced by IFNγ [39]. We therefore next examined whether the same synergy existed for MHC-I in neurons. To investigate a possible synergetic effect in N2a cells, we lowered the dose of 5-aza to 0.5 μM, and the time of IFNγ treatment to 24 h to avoid ceiling effects that could occur with the 72 h treatment. Due to this short timeframe of IFNγ treatment, we did not observe any induction of *H2* genes with IFNγ alone (Fig. 4a-c), as otherwise observed when cells were treated for 72 h (Fig. 1c-e). The low dose of 5-aza alone significantly induced *H2-D1/L* (*p* = 0.0007) (Fig. 4c), whereas neither *H2-K1* nor *H2-D1* were increased upon treatment with 5-aza alone (Fig. 4a-b). When IFNγ and 5-aza were combined, *H2-K1* gene expression was increased by 2 fold compared to untreated (*p* = 0.0007), and this increase was significantly different from both IFNγ alone (*p* = 0.0003) and 5-aza alone (*p* = 0.0023) (Fig. 4a). *H2-D1* was not induced significantly by the combined treatment with IFNγ and 5-aza (Fig. 4b). Finally, the combined treatment with IFNγ and 5-aza increased *H2-D1/L* 3.54 fold change compared to untreated (*p* = 0.001), which was significantly different compared to untreated (*p* = 0.0007), from the 2.48 fold induction observed upon treatment with 5-aza alone (*p* = 0.038), as well as from IFNγ treatment alone (*p* = 0.001) (Fig. 4c). Taken together these results revealed that blockade of DNA methyltransfer acts in synergy with IFNγ to induce expression of some but not all *H2* alleles in neurons.

We next addressed synergy between IFNγ treatment and knockdown of *DNMT1* in CGNs. We chose to focus on *DNMT1*, as knockdown of *DNMT1*, rather than *DNMT3a* or *DNMT3b*, caused an increase in *H2-D1/L*. IFNγ treatment was used as positive control for *H2* gene induction in CGNs and treatment with 0.005 ng/μl IFNγ for 72 h significantly increased *H2-D1* and *H2-D1/L* gene expression (Fig. 4d-g). Importantly, IFNγ did not increase *H2-K1* gene expression to detectable levels. We tested two different treatment combinations and timings. Either IFNγ was added simultaneously with the siRNA against *DNMT1* (Fig. 4d-e), or IFNγ was added for 72 h following treatment with siRNA against *DNMT1* (Fig. 4f-g). When IFNγ was added simultaneous with the siRNA against *DNMT1*, *H2-D1* (Fig. 4d) and *H2-D1/L* (Fig. 4e) were induced to the same extent, as when IFNγ was added in combination with non-targeting siRNA, indicating no synergy. This was in contrast to the treatment where IFNγ was added consecutive to siRNA against *DNMT1*. In this case *H2-D1/L* was induced 10.78 fold compared to untreated, which was significantly different from the 8.05 fold induction obtained upon treatment with non-targeting siRNA and then IFNγ (*p* = 0.0067) (Fig. 4g), suggesting synergy between knockdown of *DNMT1* and consecutive treatment with IFNγ.

### Knockdown of DNMT1 does not increase total MHC-I protein expression in post-mitotic mouse CGNs

**Fig. 5:**
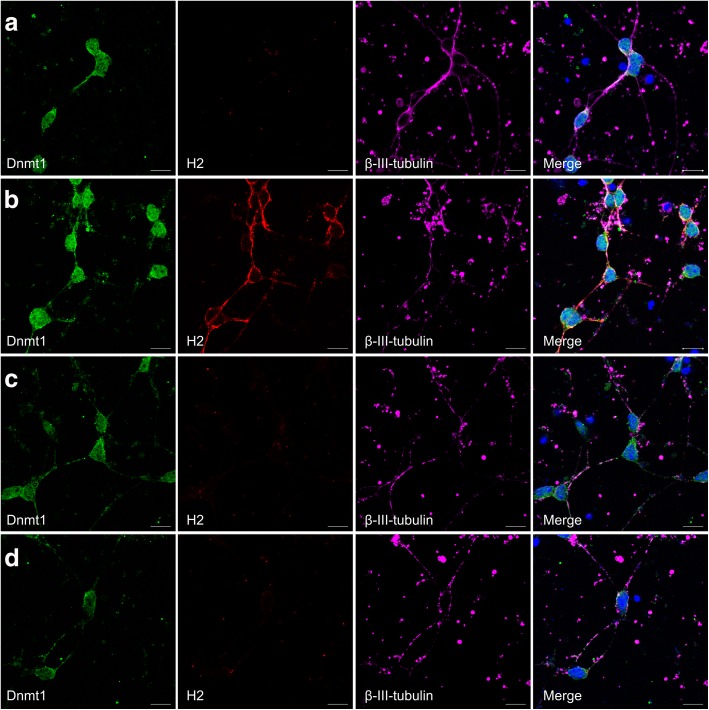
Knockdown of *DNMT1* does not increase MHC-I protein on neurons detected by immunofluorescence. DNMT1 (green), MHC-I/H2 (red), and β-III-tubulin (pink) were detected by immunofluorescence performed on untreated CGNs (**a**), CGNs treated with IFNγ (**b**), CGNs treated with non-targeting siRNA (**c**), and CGNs treated with siRNA targeting *DNMT1* (**d**). Scale bar, 10 μm

**Fig. 6:**
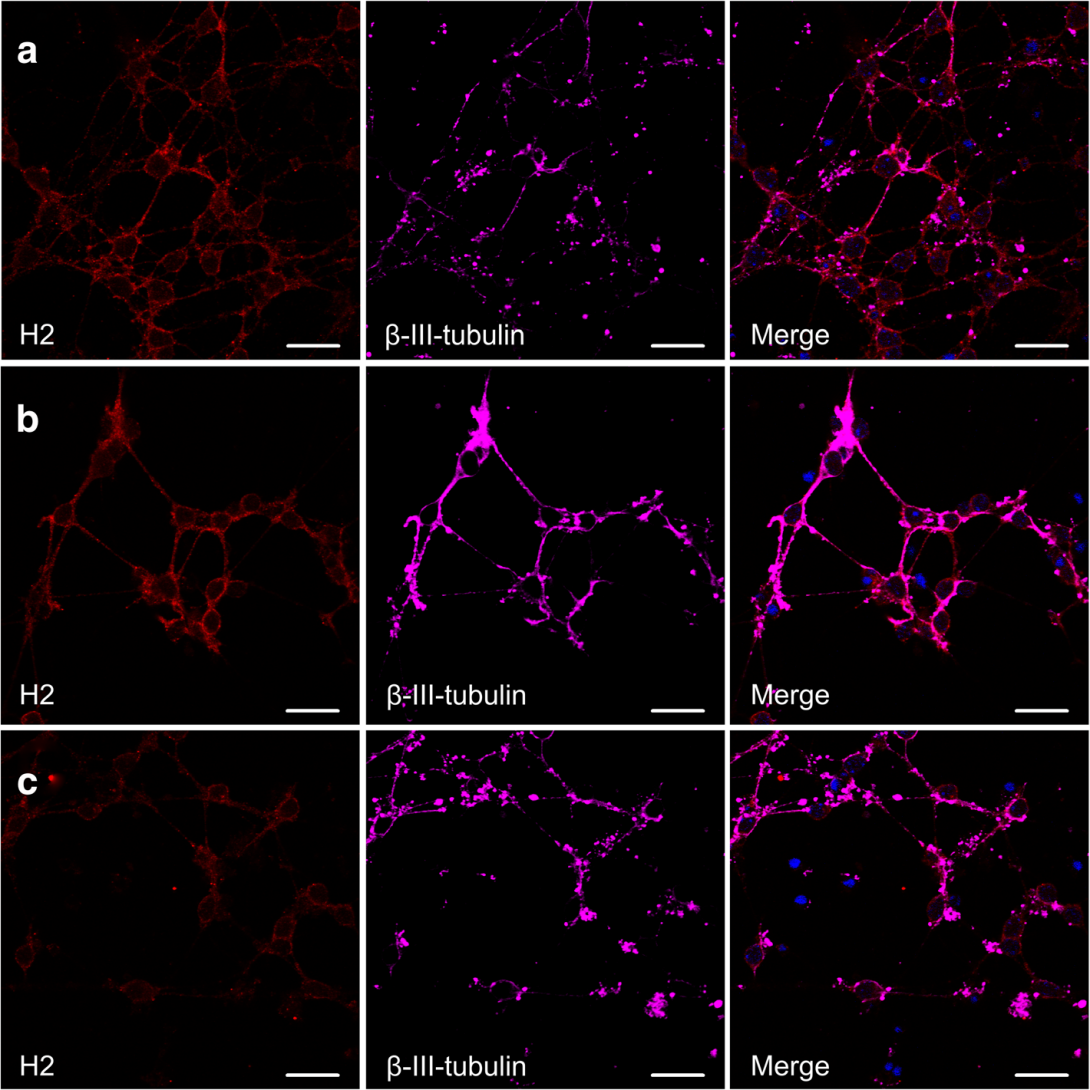
Sequential treatment with IFNγ and siRNA against *DNMT1* does not act synergistically to further increase H2 protein on neurons detected by immunofluorescence. H2 (red), and β-III-tubulin (pink) were detected by immunofluorescence performed on CGNs treated with IFNγ (**a**), CGNs treated with non-targeting siRNA and IFNγ (**b**), and CGNs treated with siRNA targeting *DNMT1* and IFNγ (**c**). Scale bar, 20 μm

**Fig. 7:**
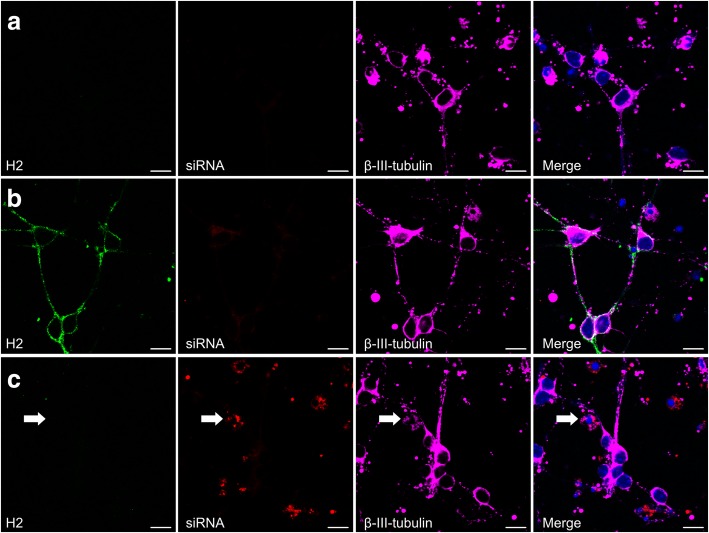
CGNs with high uptake of siRNA against *DNMT1* do not express MHC-I protein. MHC-I/H2 (green), Cy3-labeled siRNA against *DNMT1* (red), and β-III-tubulin (pink) were detected by immunofluorescence performed on untreated CGNs (**a**), CGNs treated with IFNγ (**b**), and CGNs treated with Cy3-labeled siRNA targeting *DNMT1* (**c**). Arrow indicates colocalizing Cy3-labeled siRNA targeting *DNMT1* and β-III-tubulin (neuron with high uptake of siRNA targeting *DNMT1*). Scale bar, 10 μm

We next examined protein expression of MHC-I protein upon knockdown of *DNMT1* in CGNs by immunofluorescence (Figs. 5, 6 and 7) and flow cytometry (Fig. 7). As expected, MHC-I protein was undetectable by immunofluorescence in untreated CGNs (Fig. 5a) and in CGNs treated with non-targeting siRNA for 72 h (Fig. 5c), but clearly visible on neurons upon treatment with 0.005 ng/μl IFNγ for 72 h (Fig. 5b). As *H2-K1* gene expression was not induced to detectable levels upon IFNγ treatment, the detected MHC-I protein are of the subtypes H2-D1 and/or H2-L. In our experimental group, treatment of CGNs with siRNA against *DNMT1* for 72 h did not induce MHC-I protein on the neurons (Fig. 5d).

We then addressed whether the sequential treatment of CGNs with siRNA against for *DNMT1* and then IFNγ, caused a synergistically effect on MHC-I protein levels as detected by immunofluorescence, as was the case for gene expression. We observed a clear MHC-I protein signal on the neurons treated with IFNγ (Fig. 6a), as well as for CGNs treated with non-targeting siRNA for 72 h plus additional 72 h treatment with IFNγ (Fig. 6b), but the data did not show any additional MHC-I protein upon treatment with siRNA against *DNMT1* for 72 h plus additional 72 h treatment with IFNγ (Fig. 6c). These results indicate that the synergestic effect we observed on gene expression level, does not translate into overall MHC-I protein levels, but rather shifts the balance between expressed MHC-I subtypes.

Transfection efficiency is not always complete in post-mitotic cells and can vary between the individual cells within the culture, and we therefore next aimed at investigating MHC-I protein expression by immunofluorescence on neurons that had taken up high amount of the siRNAs against *DNMT1*. We therefore transfected CGNs with a mix of the previously used SMARTpool against *DNMT1* and a Cy3-labelled single siRNA against *DNMT1*, and performed immunofluorescence for MHC-I and β-III-tubulin in conjugation with detection of the Cy3 signal from the siRNA mixture (Fig. 7). Only 3:7 siRNA molecules were Cy3-labelled to ensure that only cells that had taken up a significant amount of siRNA were visibly labelled. This approach also allowed us to use the original SMART pool in combination with the Cy3-labelled siRNA to secure consistency. Using this procedure, the Cy3-siRNA was indeed detectable on neurons (arrow), but the neurons positive for Cy3-siRNA did not display MHC-I protein (Fig. 7c), confirming our results. Untreated CGNs expressed no MHC-I protein (Fig. 7a), whereas 72 h treatment with 0.005 ng/μl IFNγ induced MHC-I protein on the neurons (Fig. 7b).

**Fig. 8:**
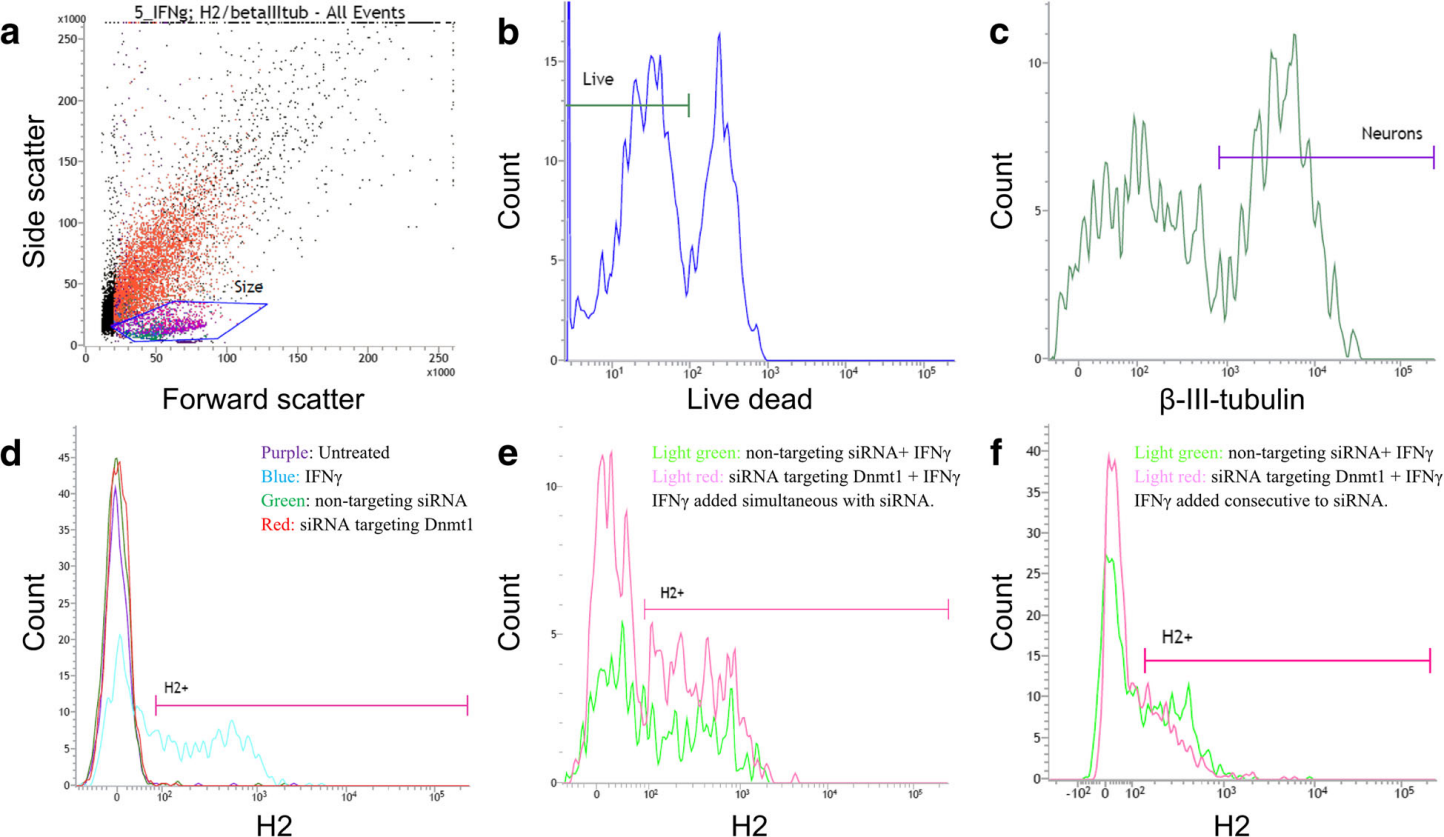
Knockdown of *DNMT1* does not increase total MHC-I protein level on neurons detected by flow cytometry. CGN cultures were harvested and processed for flow cytometry, and analyzed the same day. Gating strategy is shown in A-C; a population experimentally determined to contain neurons were gated based on size (**a**), and from this population we proceeded with the live cells (**b**), from which the β-III-tubulin positive cells (neurons) were gated for further studies (**c**). D-F show MHC-I histograms for experimental groups gated on the neuronal population. Whereas treatment with IFNγ (blue) caused MHC-I expression on neurons, no MHC-I positive neurons were detected in untreated CGNs (purple), CGNs treated with non-targeting siRNA (green), or in CGNs treated with siRNA against *DNMT1* (red) (**d**). Histograms of MHC-I signal in Fig. 8E-F show overlay of CGNs treated with non-targeting siRNA and IFNγ (light green), and CGNs treated with siRNA against *DNMT1* and IFNγ (pink), and Fig. 8E shows the results from the cells receiving siRNA and IFNγ simultaneously, whereas Fig. 8F shows the results from the cells receiving IFNγ after the siRNA

We supplemented the immunofluorescence with flow cytometry studies, to be able to examine all neurons in the culture in a systematic way (Fig. 8). We gated the neurons by size (Fig. 8a), and viability (Fig. 8b), and then analyzed the β-III-tubulin-positive population (Fig. 7c) for extracellular MHC-I protein expression (Fig. 8d). Histogram of MHC-I signal shows overlay of untreated CGNs (purple), CGNs treated with non-targeting siRNA (green), CGNs treated with siRNA against *DNMT1* (red) and CGNs treated with 0.005 ng/μl IFNγ for 72 h (blue) (Fig. 8d). IFNγ treatment induced a MHC-I + population of neurons (49%) with a median fluorescence intensity of 360, whereas treatment of CGNs with siRNA against *DNMT1* did not induce extracellular MHC-I on the neurons (Fig. 8d) confirming our immunofluorescence data. We next addressed whether synergy between knockdown of *DNMT1* and IFNγ existed on the MHC-I protein level. Histograms of MHC-I signal in Fig. 8e-f show overlay of CGNs treated with non-targeting siRNA and IFNγ (light green), and CGNs treated with siRNA against *DNMT1* and IFNγ (pink), and Fig. 8e shows the results from the cells receiving siRNA and IFNγ simultaneously (like Fig. 4d-e), whereas Fig. 8f shows the results from the cells receiving IFNγ after the siRNA (like Fig. 4f-g and Fig. 6). The percentage of neurons displaying MHC-I signal, as well as the median fluorescence intensity of the PE- MHC-I signal, were in both instances comparable between the cells receiving non-targeting siRNA and IFNγ, and the cells receiving siRNA against *DNMT1* and IFNγ, indicating no synergy with regard to total MHC-I protein level.

## Discussion

In contrast to other nucleated cells, most post-mitotic neurons do not express MHC-I under non-pathological conditions [13, 14]. However, during several pathological conditions MHC-I expression can be induced in neurons where they are believed to play an active role in the disease process. This is the case for both autoimmune diseases, where neurons can be killed by CD8^+^ T cells [24–26], and for neurodegenerative disorders [5, 6]. Knowledge on the molecular mechanisms regulating neuronal MHC-I might thus be of relevance in a range of CNS diseases. We chose to address whether MHC-I expression on neurons is regulated by DNMT1. DNMT1 is traditionally thought of as the maintenance DNA methyltransferase during cell division, but today we know that DNMT1 mRNA and protein is expressed in post-mitotic neurons [30, 31], suggesting a more complex role. It was also previously believed that methylation patterns were stable in post-mitotic neurons, but it is now clear that active demethylation is an ongoing process that in neurons is tightly regulated by multiple enzymes including the ten eleven translocation (TET) enzyme TET1 [40–42], and Gadd45b (growth arrest and DNA-damage-inducible 45β) which is induced upon neuronal stimulation [43].

MHC-I expression is controlled by methylation in tumour cell lines and human PBMCs [44, 45]. We here show that this is also the case in neuron-like cell cultures, by showing that two inhibitors of DNA methylation (PCA and 5-aza) increased the expression of genes encoding MHC-I in human and mouse neuroblastoma cell lines. This effect was observed upon 72 h of incubation with the compounds, and, as we assume that the cells have divided in this timespan, this effect most likely is dependent on the passive demethylation process of dividing cells.

Importantly, we next examined non-dividing post-mitotic CGNs. Here we show that a decrease in *DNMT1* specifically causes an increase in *H2-D1/L* gene expression, while *H2-K1* gene expression was still undetectable after *DNMT1* knockdown. Importantly, knockdown of *DNMT3a* and *DNMT3b* did not change *H2* levels. This suggests that DNMT1 does indeed have a more complex role in post-mitotic neurons than merely being the maintenance DNA methyltransferase.

The increase in MHC-I mRNA expression we observed was not sufficient to cause an upregulation of MHC-I protein on the surface. For MHC-I protein to be expressed on the surface, the MHC-I chain needs to form a stable complex with β2M [46]. *β2M* baseline expression was high in CGNs and did not change with *DNMT1* knockdown, we therefore suspect that β2M is not the rate limiting step for MHC-I surface expression. Instead additional stimulation is likely needed for MHC-I to be expressed on the surface. It has for example been shown that TAP1 and TAP2 expression is needed for surface expression of MHC-I [47, 48]. The relevance of additional factors has been suggested before in a study of cultured rat hippocampal neurons [49]. The authors showed that 40% of untreated neurons expressed MHC-I transcripts, but only 10% of the neurons expressed β2M mRNA, and none expressed TAP1 and TAP2 mRNA [49]. Interestingly in the same culture system, MHC-I was upregulated not only by IFNγ but also in neurons that had been electrically silenced by treatment with tetrodotoxin (TTX) [14]. Upregulation of the entire machinery for MHC-I expression, has also been shown to occur with aging [50].

We suggest, that by knocking down DNMT1 we create a situation where H2-D1 and H2-L, but not *H2-K1* becomes more accessible for the translational machinery. This will increase the baseline transcription of the gene slightly, as we see in our data. The effect is however small and confined to the mRNA level. For MHC class I protein to be expressed more factors such as β2M, TAP1 and TAP2 are needed. Changes in DNMT1 levels thus only affects which H2-genes are expressed but it does not affect the total rate of protein expression.

If what we suggest is true, and lowering DNMT1 levels indeed causes the H2-D1/L loci to become more accessible, then we should be able to predict that any stimulus that induces MHC class I protein expression would induce even more H2-D1/L expression in cells with lowered DNMT1. This was indeed the case. We first confirmed that MHC-I protein on the surface of neurons can be induced by IFNγ alone as previously reported [13, 14], and in addition we showed that in CGNs, IFNγ treatment only induce *H2-D1* and *H2-D1/L* gene expression and not *H2-K1* gene expression. When stimulating the CGNs with IFNγ after *DNMT1* knockdown, *H2-D1/L* expression was increased by 10.78 fold compared to untreated CGNs, in contrast to 8.05 fold in CGNs treated with non-targeting siRNA. The same synergistic effect was observed in N2a neuroblastoma cell line, where combined treatment with IFNγ and 5-aza led to higher gene induction of *H2-D1/L* gene expression than the treatment with either one individually. In contrast to CGNs, *H2-K1* was also induced to a higher extent by IFNγ in N2a cells following 5-aza treatment. Synergy between changes in methylation and IFNγ signaling has been shown before, in a previous study showing that IFNγ and the demethylating agent zebularine acted synergistically to increase the gene expression of *IDO1* [39].

Non-targeting siRNA across several experiments consistently induced *H2-D1* gene expression or interfered with its detection for unknown reasons, and it was thus not possible to distinguish if the *H2-D1* induction upon knockdown of *DNMT1* was due to lower levels of *DNMT1*, or just the mere presence of a siRNA. However, the same effect was not observed upon introduction of siRNA against *DNMT3a* or *DNMT3b*, suggesting that *H2-D1* does indeed increase upon *DNMT1* knockdown, and that the signal with non-targeting siRNA was due to a technical problem with the probes. The probe detecting both *H2-D1* and *H2-L* (*H2-D1/L* collectively) was not influenced by non-targeting siRNA supporting this idea. With current available probes we cannot distinguish the signals from *H2-D1* and *H2-L*.

Our results consistently show that knockdown of *DNMT1* does not cause an increase of total MHC-I protein in neurons, not even in the neurons displaying the highest uptake of siRNA against *DNMT1*. Two different antibodies and techniques were used, strengthening the credibility of the result. For flow cytometry studies we investigated surface MHC-I, and the possibility that intracellular MHC-I protein was increased without being translocated to the cell surface thus exists, however in that case we would have expected to observe an intracellular MHC-I signal in immunofluorescence. Another issue is that the antibodies used for immunofluorescence and flow cytometry are not subtype-specific antibodies, but rather were chosen based on their previously reported performance in immunofluorescence and flow cytometry [23, 51].

It would be interesting to investigate MHC-I protein using subtype-specific antibodies, but we were not able to obtain a sufficient signal-to-noise ration with the currently available subtype-specific MHC-I antibodies.

Our data suggests that DNMT1, when present in neurons, can inhibit expression of the *H2-D1/L* loci. Whether this is through a direct effect on methylation status of the *H2-D1* or *H2-L* loci, or through some other mechanism, is still unknown. The mechanism could also be indirect through changes in the transcriptional machinery induced by the lack of DNMT1 activity. Interestingly, we observe that this effect of DNMT1 does not affect all *H2* loci to an equal extend. Since we at the same time did not observe any changes in total surface expression of MHC-I protein, this suggests that it might instead shift the balance of available MHC-I protein from one subtype to another. This could be highly important in the context of autoimmunity, where epitope presentation to T-cells depend on the MHC-I subtype. The phenomenon of differential response of the different MHC-I subtypes have been reported before in peripheral blood leukocytes, colon mucosa, and larynx mucosa, with some MHC-I subtypes having a lower constitutive expression and being more inducible [52, 53]. This could also play an important role in the development of neurodegenerative diseases such as ALS. It has been shown in a mouse model of ALS, that increasing MHC class I expression on motor neurons protects the neurons against astrocyte toxicity [6]. The result was that the mice survived longer and had a better motor performance. Interestingly, this effect was only seen with *H2-K* and not *H2-D*. This could perhaps be attributed to the fact that *H2-D1* is inhibited by the presence of DNMT1 in mature motor neurons while H2-K is not.

DNMT1 have been studied in post-mitotic neurons by others. DNMT1 has for example been found to colocalize with GAD67-positive GABAergic neurons in many parts of the brain [54]. Curiously, Fan et al. found no effect of postnatal brain-specific knockdown of *DNMT1*, neither on global methylation levels nor on neuronal long-term survival [31]. The simultaneous gene deletion of *DNMT1* and *DNMT3a* however, affected synaptic function, decreased learning and memory formation, and caused upregulation of MHC-I and *STAT1* gene expression in adult mouse hippocampus [55]. It might seem contradictory that post-mitotic cells would depend on continuous methylation, however with the discovery of an oxidized variant of 5mC, the 5-hydroxymethylcytosine (5hmC), which was discovered simultaneously in mouse ESCs [42] and in adult mouse cerebellar Purkinje cells [56], the concept of active demethylation arose. Methylated DNA can be demethylated without cell division, when 5hmC is converted to 5C through a series of events involving the TET enzymes and base excision repair pathways [40, 42]. Active demethylation is stimulated by neuronal activity acting through TET1 and Gadd45b [40–43]. For active demethylation to occur, and thus for the neurons to depend on re-methylation by DNMT1, it might be a prerequisite that the neurons have formed an active network. CGNs are glutamatergic but receive mostly inhibitory inputs from GABA and glycine containing synapses. This cannot be reproduced in vitro, and this might be an important limitation in our study. It is also possible that such inherent properties of the neuronal cultures vary from one preparation of CGNs to another. In light of this, it would be interesting to study knockdown of *DNMT1* in other in vitro models, such as organotypic slice cultures, or in combination with induction of active demethylation. We do detect synaptic markers in our CGN cultures, and interestingly observe a strong effect of DNMT1 downregulation on these synaptic markers. This is particularly interesting as MHC-I are known to be involved in synaptic plasticity. It is tempting to speculate that lack of DNMT1 causes an upregulation of MHC-I, which in turn causes elimination of synapses in the CGN culture. The MHC-I surface protein would be removed in this process explaining why we do not see it. However, from our present data we can not say anything about the direction of the correlation between increased MHC-I expression and lower expression of synaptic markers. It could also be that DNMT1 plays an important role in controlling the transcriptional processes related to synapse maintenance, and that the upregulation of MHC-I is secondary to this effect. DNA methylation status has indeed been show to control transcription-dependent regulation of glutamatergic synaptic homeostasis [57].

In conclusion we have shown that MHC-I gene expression is regulated by DNMT1 in human and mouse neuroblastoma cell lines, and that the gene expression of some but not all *H2* subtypes is upregulated in post-mitotic CGNs upon knockdown of *DNMT1*. We moreover show that IFNγ can act in synergy with these treatments to further increase *H2-D1/L* gene expression. Importantly, this effect differs between *H2* subtypes.

## Methods

### SK-N-AS cell culturing and addition of procainamide and IFNγ

The human neuroblastoma cell line SK-N-AS was obtained from ATCC. SK-N-AS cells were cultured in a 50/50 mixture of Ham’s F-12 media (Gibco, Life Sciences), and Iscove’s Modified Dulbecco’s Medium (IMDM) (Biowest, Nuaillé, France), supplemented with 10% Fetal calf serum (FCS) (Sigma-Aldrich, St. Louis, MO, USA), and 0.5% penicillin-streptomycin (P/S) (Lonza Ltd., Basel, Switzerland). SK-N-AS cells were seeded at a density of 3 × 10^4 cells/ml at day 1 in Ham’s F-12/IMDM containing 10% FCS and 0.5% P/S, then on day 2 media was changed to Ham’s F-12/IMDM containing 2% FCS and procainamide (PCA) in the dose range 0.1–2 mM or 0.02 ng/μl IFNγ was added to the cells. At day 5 the cells were harvested for RNA extraction.

### siRNA treatment of SK-N-AS cells

SK-N-AS cells were seeded at a density of 75 × 10^4 cells/ml at day 1 in Ham’s F-12/IMDM containing 10% FCS and 0.5% P/S. On day 2 media was changed to Ham’s F-12/IMDM without FCS and P/S containing instead 15 nM siRNA and Lipofectamine 2000 (Life Technologies). The siRNAs used were Ambion™ Silencer™ Select siRNA against human *DNMT1* (s4215), *DNMT3a* (s200426) and *DNMT3b* (s4221). After 5 h of transfection, the media was changed to Ham’s F-12/IMDM containing 10% FCS and 0.5% P/S and the cells were incubated for 72 h at 37 °C before harvest and RNA extraction.

### N2a cell culturing and addition of 5-aza-2-deoxycytidine and IFNγ

The murine neuroblastoma cell line N2a was obtained from ATCC. Undifferentiated N2a cells were cultured in Dulbecco’s Modified Eagle Medium (DMEM) high glucose (Biowest, Nuaillé, France), supplemented with 10% Fetal calf serum (FCS) (Sigma-Aldrich, St. Louis, MO, USA), and 0.5% penicillin-streptomycin (Lonza Ltd., Basel, Switzerland). N2a cells were seeded at a density of 4.5 × 10^4 cells/ml at day 1 and 0.5 ng/μl IFNγ was added on day 2. 5-aza-2-deoxycytidine (5-aza) was added on day 2, 3, and 4 in the doses 5 μM and 80 μM, and at day 5 the cells were harvested for RNA purification. The 5-aza treatment was renewed every 24 h, due to the short half-life of this compound. When addressing possible synergy between 5-aza treatment and IFNγ treatment, the same protocol for 5-aza treatment was followed, but with the addition of 0.05 ng/μl IFNγ at day 4 and with a lower concentration of 5-aza (0.5 μM).

### Preparation and culturing of primary cultures of cerebellar granular neurons

Primary CGN cultures were prepared from 7 to 9 BALB/cJBomTac (Taconic, Denmark) pups at postnatal day six, essentially as described by Schousbo et al. [58]. Cerebellum was isolated from a maximum of two pups at a time, the meninges removed on ice in a dissection buffer containing Krebs-Ringer buffer (Substrate-department, Faculty of Health Science, University of Copenhagen), Bovine Serum Albumin (BSA) (Sigma-Aldrich, St. Louis, MO, USA), MgSO_4_ (RegionH Apoteket), and HEPES buffer (Gibco, Life Sciences), and the remaining tissue was mechanically and enzymatically homogenized by chopping and trypsination. Cells were then washed in a buffer containing trypsin inhibitor and DNase I (Sigma-Aldrich, St. Louis, MO, USA), centrifuged briefly at 100 rpm to stratify cells, and the uppermost layers were transferred to Krebs-Ringer buffer supplemented with BSA (Sigma-Aldrich, St. Louis, MO, USA), MgSO_4_ (RegionH Apoteket), HEPES (Gibco, Life Sciences), CaCl_2_ (RegionH Apoteket). Upon centrifugation for 10 min. at 700 rpm, the pelleted cells were resuspended in NBM-A (Gibco, Life Sciences) supplemented with B27 (Gibco, Life Sciences), BSA (Sigma-Aldrich, St. Louis, MO, USA), Glutamax (Gibco, Life Sciences), and HEPES (Gibco, Life Sciences). Cells were seeded at a density of 4 × 10^5 cells/ml in 24-well plates coated with poly-D-lysine (Sigma-Aldrich, St. Louis, MO, USA), and incubated at 37 °C, 5% CO_2_. 24 h after seeding of cells, cytosine β-D-arabinofuranoside hydrochloride (Ara-C) (Sigma-Aldrich, St. Louis, MO, USA) was added to the media to a final concentration of 10 μM to inhibit the growth of glial cells.

### siRNA for primary cultures of cerebellar granular neurons

The siRNAs against mouse *DNMT1*, *DNMT3a* and *DNMT3b* were bought as a pool of four siRNAs targeting the same gene in different sites, called SMARTpool, from the Accell range by Dharmacon™, part of GE Healthcare, Little Chalfont, Buckinghamshire, United Kingdom (See Additional file 1: Table S1). Negative control siRNA, “Accell Non-targeting #1” was designed, modified and microarray-confirmed by Dharmacon™ to have minimal targeting of known genes in mouse cells. The siRNAs were diluted in the supplied 5XsiRNA buffer diluted in RNase-free water, according to the manufacturer’s instructions, aliquoted and stored at − 20 °C.

### Addition of IFNγ and siRNA to primary cultures of cerebellar granular neurons

On the day of siRNA transfection the normal growth media of CGNs was exchanged with preheated and CO_2_-equilibrated NBM-A media supplemented with B27, Glutamax and HEPES containing 10 μM Ara-C and 1 μM siRNA, but excluding BSA. Transfection efficiency was assessed using a Cy3-labelled non-targeting siRNA (Additional file 1: Figure S1). For studies of the effect of siRNA on *H2* and *DNMT* genes (Figs. 3 and 4), the effect of siRNA on MHC-I protein by immunofluorescence (Figs. 5 and 7), and the effect of siRNA on H2 protein by flow cytometry (Fig. 8), CGNs were treated with 1 μM siRNA SMARTpool or non-targeting siRNA control for 72 h prior to harvesting or fixation for further processing. For detection of the neurons receiving the SMARTpool against *DNMT1* by immunofluorescence (Fig. 7), the SMARTpool against *DNMT1* was mixed 7:3 with a custom-made Cy3-labelled siRNA against *DNMT1*, and the mix was then added to cultures in equimolar amount with regards to the SMARTpool against *DNMT1* as was used in previous experiments. In these experiments, treatment with 0.005 ng/μl IFNγ for the same 72 h prior to processesing was used as a positive control for H2 induction. When testing synergy between *DNMT1* knockdown and IFNγ treatment, two different protocols were tested. Either 0.005 ng/μl IFNγ was added on the same day as the siRNAs, followed by RNA extraction 72 h later (Fig. 4d-e). Alternatively cells were first subjected to 72 h of siRNA treatment, then media was exchanged to normal growth media including 10 μM Ara-C and BSA, and 0.05 ng/μl IFNγ was added to the cells, which were then incubated for another 72 h before harvest for RNA extraction (Fig. 4f-g) or fixation for immunofluorescence (Fig. 6).

### Quantification of gene expression

Cells were harvested in RLT buffer on ice, and RNA was purified using RNeasy® Mini Kit (Qiagen, Hilden, Germany) according to the manufacturer’s instruction for harvesting animal cells. The quality and concentration of the purified RNA was determined by measuring optical density (OD, 260/280) on a NanoDrop 2000c Spectrophotometer (ThermoFischer Scientific, Waltham, MA USA). The purified RNA was used to generate cDNA using QuantiTect® Reverse Transcription Kit, which includes an enzymatic removal of all genomic DNA (Qiagen, Hilden, Germany). Real-time quantitative PCR was performed in an Applied Biosystems Quant Studio™ 7 Flex station (Life Technologies™, Applied Biosystems, Foster City, CA, United States). The PCR primers and FAM-conjugated TaqMan® Gene Expression Assays probes (Additional file 1: Table S2) were from LifeTechnologies (Applied Biosystems, Foster City, CA, United States). The real-time PCR was carried out with TaqMan® Gene Expression Master Mix (Applied Biosystems, Foster City, CA, United States), which contained AmpliTaq® Gold DNA Polymerase, AmpErase, UNG, dNTPs with dUTP, and optimized buffer components. The thermal cycle conditions were: part A: 50 °C for 2 min to activate the polymerase, part B: 95 °C for 10 min, part C: 95 °C for 15 s for denaturation, and part D: 60 °C for 1 min for annealing and extension, repeating part C and D for 45 cycles. We assessed the degree and specificity of the knockdown of targeted genes; *DNMT1*, *DNMT3a* and *DNMT3b* using real time quantitative PCR (Figs. 2 and 3). Evaluation of an actual functional effect of the knockdown was performed by measuring if the gene expression of positive control genes *NNAT* and *S100A10* were upregulated. The two genes *NNAT* and *S100A10* have previously been reported to be upregulated upon knockdown of *DNMT1* in a neuronal cell line [59]. For details, see Additional file 1: Figure S2. MHC-I gene expression was evaluated using probes against *HLA-A*, *HLA-B* and *HLA-C* in human SK-N-AS neuroblastoma cell line, and for mouse cells using probes against β2-microglobulin (*β2M)*, *H2-D1*, *H2-K1* and a probe detecting *H2-D1* and *H2-L* (*H2-D1/L*) as it is not possible to choose TaqMan probes specifically for *H2-L*. Endogenous expression of *ACTB* and *GAPDH* was measured and used to calculate relative expression level changes between samples. The signal obtained from *HLA-C* in SK-N-AS cells, and from *H2-K1* in CGNs was undetectable (Ct (*HLA-C*) > 35, Ct (*H2-K1*) > 40), and was not further analyzed.

### Immunofluorescence

CGNs were seeded on Nunc™ LabTek™ chamber slides (ThermoFischer Scientific, Waltham, MA, USA), coated with poly-D-lysine (Sigma-Aldrich, St. Louis, MO, USA). Cells were either left untreated, or treated with 0.005 ng/μl IFNγ, or transfected with (A) non-targeting siRNA, (B) SMARTpool against *DNMT1* or (C) mix of SMARTpool against *DNMT1* and Cy3-labelled siRNA against *DNMT1*, in NBM-A media as described above, and after 72 h of incubation fixated in 4% paraformaldehyde (Electron Microscopy Sciences, Hatfield, PA, United States). Cells were incubated 20 min at room temperature in blocking buffer (PBS containing 2% BSA (Sigma-Aldrich, St. Louis, MO, USA), 0.3% TritonX-100 (AppliChem, Darmstadt, Germany), and 5% Normal Goat Serum (Dako, Glostrup, Denmark)). Primary antibodies diluted in blocking buffer were added after blocking, and incubated overnight at room temperature. Primary antibodies were; rat monoclonal IgG2a anti-mouse MHC class I antibody (1:200, clone ErHr52, BMA Biomedicals, Augst, Switzerland), mouse monoclonal IgG1 anti-β-III-tubulin antibody (1:50, clone TUJ-1, Santa Cruz, Texas, USA), and rabbit polyclonal anti-DNMT1 antibody (NB100–264, Novus Biologicals, CO, USA). After three washing steps in PBS, fluorophore-conjugated secondary antibodies diluted in blocking buffer were added, and incubated for 1 h at room temperature. Secondary antibodies were; Alexa Fluor® 647 goat anti-mouse (1:2000), Alexa Fluor® 555 goat anti-rat (1: 2000), Alexa Fluor® 488 goat anti-rat (1: 2000) and Alexa Fluor® 488 goat anti-rabbit (1:1000) (all Life Technologies, OR, USA). 4′,6-Diamidino-2-Phenylindole, Dilactate (DAPI) (Life Technologies, OR, USA) diluted 1:30.000 in PBS was added to the cells immediately upon incubation with secondary antibody, and incubated 8 min at room temperature. Following three washing steps in PBS, and one quick rinse in milliQ water, the slides were mounted with ProLong® Antifade Gold mounting media (Life Technologies, OR, USA). Immunofluorescence images were taken with a Zeiss LSM510 confocal scanning microscope, and analyzed using the Carl Zeiss imaging software Zen Black.

### Flow cytometry

CGNs were at day 5 in vitro (DIV5) left untreated, transfected with non-targeting siRNA or with *DNMT1* SMARTpool, or treated with 0.005 ng/μL IFNγ. At DIV8 cells were harvested by brief trypsinization (2 min, 37 °C) in NBM-A containing BSA, transferred to 5 ml Falcon tubes for antibody staining, and pelleted at 400 RPM, 5 min, 4 °C. Staining was performed as follows with washes in in 3 ml PBS (Biowest, Nuaillé, France) between each step. First, cells were stained for live/dead cells for 30 min at 4 °C using the Fixable Viability Dye eFluor 506 (eBioscience, Aarhus, Denmark) diluted in PBS. Blocking of unspecific signal was done by incubation for 20 min on ice in 10% FCS (Merck Millipore, Berlin, Germany) diluted in PBS. Staining was performed for 20 min on ice with PE-conjugated rat IgG2a,_κ_ anti-mouse MHC-I antibody (clone M1/42, 125,505, BioLegend, Copenhagen, Denmark) or PE-conjugated rat IgG2a,_κ_ isotype control (clone RTK2758, 400,507, BioLegend, Copenhagen, Denmark), both diluted to 1 μg/ml PBS containing 2% FCS. If needed, cells were fixated in PBS containing 2% paraformaldehyde (Electron Microscopy Sciences, Hatfield, PA, United States) for 15 min at room temperature on a shaker and permeabilized in PBS containing 0.7% Tween20 (Bio-Rad, CA, USA) for 15 min at room temperature on a shaker. Intracellular staining was performed over 30 min at room temperature on a shaker with Alexa Fluor 647-conjugated mouse IgG2a,_κ_ anti-β-III-tubulin antibody (clone AA10, 657,405, BioLegend, Copenhagen, Denmark) or Alexa Fluor 647-conjugated mouse IgG2a,_κ_ isotype control (clone MOPC-173, 400,239, BioLegend, Copenhagen, Denmark), both diluted to 0.5 μg/ml in PBS containing 0.5% Tween20, 1% BSA (Sigma-Aldrich, St. Louis, MO, USA) and 10% Donkey Normal Serum (DAKO, Glostrup, Denmark). Finally, cells were resuspended in either PBS containing 2% FCS (extracellular staining only, cells not fixated) or in PBS (intracellular staining, cells fixated) and run on a FACSVerse flow cytometer (BD Biosciences, NJ, USA) equipped with FACSuite software for data analysis.

### Statistics

The expression of the targeted gene was calculated using the ∆∆Ct method, relative to expression levels of the housekeeping genes *ACTB* and *GAPDH*. Data are presented as mean fold change with upper and lower intervals calculated as fold change +/− standard error of the mean (S.E.M.). Statistical significance was tested using One-Way or Two-Way ANOVA based on the ∆∆Ct values with a Dunnett’s multiple comparison test. The *p* values reported are corrected for multiple comparisons. For testing the effect of treatments, a One-Way ANOVA with a Dunnett’s multiple comparison test against untreated or non-targeting siRNA was used. For testing synergy between two treatments, a Two-Way ANOVA with a Dunnett’s multiple comparison test against the combined treatment, was used.

## Additional file

Additional file 1: **Figure S1.** Transfection efficiency of Cy3-labelled siRNA in CGNs. CGNs were transfected with Cy3-labelled non-targeting siRNA as described in Materials and Methods. After 72 h incubation the cells were fixated and mounted using mounting media containing DAPI. Epifluorescence images were obtained for DAPI, Cy3-siRNA and overlay. Arrows with thick arrowhead, transfected cell; thin arrowhead, non-transfected cell. **Figure S2.** Generation of a functional positive control of DNMT1 knockdown. (A) Gene expression of NNAT, CD24A, ICAM1, RUNX1, and S100A10 [46] in CGNs upon knockdown of DNMT1 relative to untreated cells. Bonferroni-corrected one-sample t-test: * p1.5 for NNAT and S100A10 relative to treatment with non-targeting siRNA. (C) Positive control values for three successful and four unsuccessful experiments (mean ± SD). **Table S1.** siRNAs from DharmaconTM used in the study. **Table S2.** Taqman probes used in the study. (DOCX 930 kb)

## Abbreviations

ADCA-DN: Autosomal dominant cerebellar ataxia, deafness and narcolepsy
CGNs: Cerebellar granule neurons
DNMT1: DNA methyltransferase 1
MHC: Major Histocompability Complex
MHC-I: MHC class I
