# Supplementary material for: DNMT1 regulates expression of MHC class I in post-mitotic neurons
Source: Mol Brain. 2018 Jul 3;11:36. doi: 10.1186/s13041-018-0380-9 (PMC6029374; doi:10.1186/s13041-018-0380-9)
Supplement: Supplementary file 1 — Figure S1. Transfection efficiency of Cy3-labelled siRNA in CGNs. CGNs were transfected with Cy3-labelled non-targeting siRNA as described in Materials and Methods. After 72 h incubation the cells were fixated and mounted using mounting media containing DAPI. Epifluorescence images were obtained for DAPI, Cy3-siRNA and overlay. Arrows with thick arrowhead, transfected cell; thin arrowhead, non-transfected cell. Figure S2. Generation of a functional positive control of DNMT1 knockdown. (A) Gene expression of NNAT, CD24A, ICAM1, RUNX1, and S100A10 [46] in CGNs upon knockdown of DNMT1 relative to untreated cells. Bonferroni-corrected one-sample t-test: * p1.5 for NNAT and S100A10 relative to treatment with non-targeting siRNA. (C) Positive control values for three successful and four unsuccessful experiments (mean ± SD). Table S1. siRNAs from DharmaconTM used in the study. Table S2. Taqman probes used in the study. (DOCX 930 kb) [file 13041_2018_380_MOESM1_ESM.docx]

Additional file 1

**
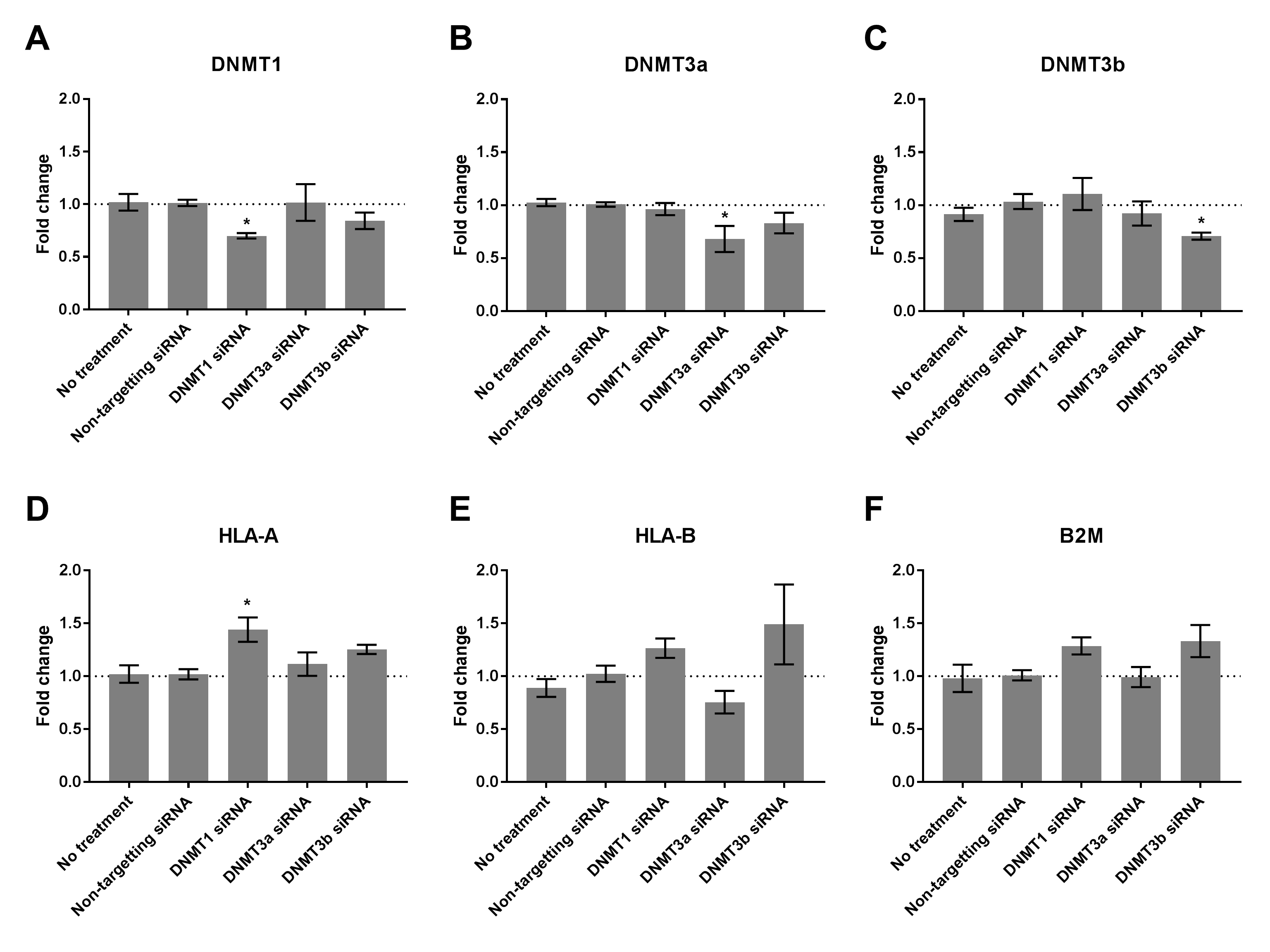
**

**Figure S1. Knockdown of *DNMT1* increases MHC-I gene expression in a human neuroblastoma cell line.**

Shown is the fold change in gene expression of *DNMT1* (A), *DNMT3a* (B), *DNMT3b* (C), *HLA-A* (D), *HLA-B* (E), and *β2M* (F) following 72h siRNA treatment of SK-N-AS cell cultures. Baseline indicate gene expression level in cells treated with non-targeting siRNA. Bars represent mean +/- S.E.M., N = 4-7 from three independent experiments. Stars illustrate effects that are significantly different from non-targeting siRNA treatment. * *p* < 0.05, multiplicity corrected p-values in One-Way ANOVA with Dunnett’s post hoc test.

**
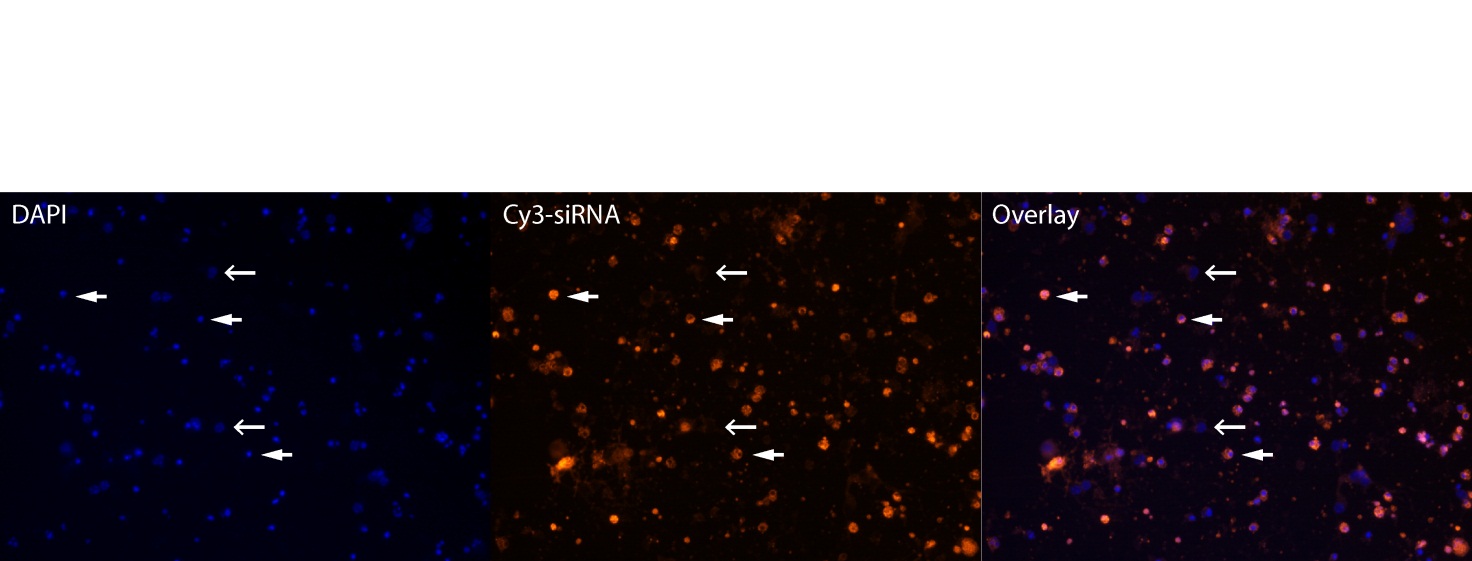
**

**Figure S2. Transfection efficiency of Cy3-labelled siRNA in CGNs**

CGNs were transfected with Cy3-labelled non-targeting siRNA as described in Materials and Methods. After 72 hrs incubation the cells were fixated and mounted using mounting media containing DAPI. Epifluorescence images were obtained for DAPI, Cy3-siRNA and overlay. Arrows with thick arrowhead, transfected cell; thin arrowhead, non-transfected cell.


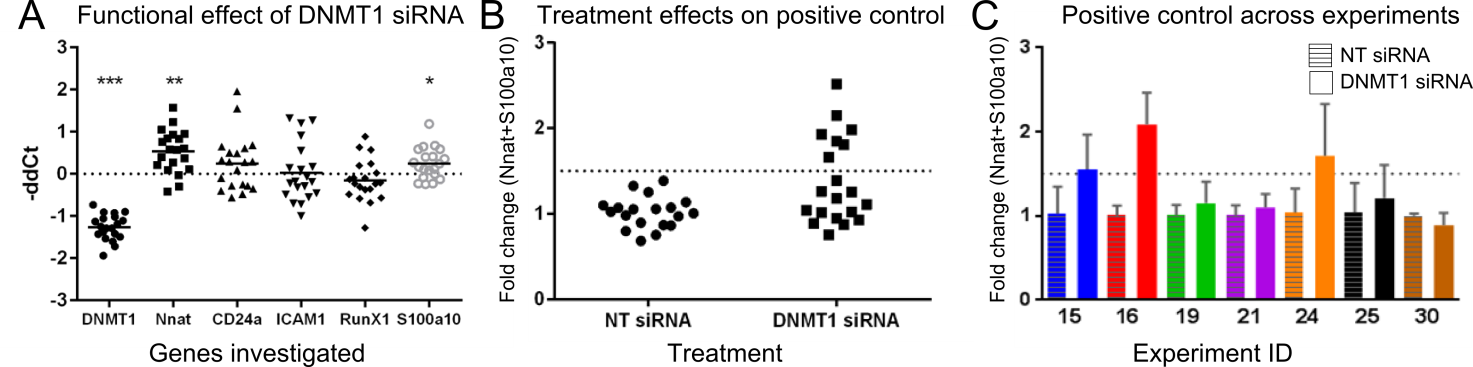


**Figure S3. Generation of a functional positive control of *DNMT1* knockdown**

Gene expression of *NNAT*, *CD24A*, *ICAM1*, *RUNX1*, and *S100A10* [40] in CGNs upon knockdown of *DNMT1* relative to untreated cells. Bonferroni-corrected one-sample t-test: * p<0.05, ** p<0.01, and *** p<0.001. (B) As positive control for functional downregulation of DNMT1 we used additive fold change of >1.5 for *NNAT* and *S100A10* relative to treatment with non-targeting siRNA. (C) Positive control values for three successful and four unsuccessful experiments (mean±SD).

**Table S1. siRNAs from DharmaconTM used in the study.**

| **Target** | **Catalogue number** | **siRNAs in SMARTpool** | **Target Sequence** |
| --- | --- | --- | --- |
| Non-targeting siRNA #1 | D-001910-01-20 | only one siRNA | UGGUUUACAUGUCGACUAA |
| Mouse *DNMT1* SMARTpool | E-056796-00-0020 | A-056796-13 | CCAUUGGCCUGGAGAUUAA |
|  |  | A-056796-14 | GGAUGAACCCCAGAUGUUG |
|  |  | A-056796-15 | GUAUGAGGUUUGUUUUGUG |
|  |  | A-056796-16 | GUCUUGUGCUCAGUGUCUG |
| Mouse *DNMT3a* SMARTpool | E-065433-00-0010 | A-065433-13 | UUCUCGACUCCAGAUGUUC |
|  |  | A-065433-14 | CGUGUAAGUGUGAAGAUUU |
|  |  | A-065433-15 | GCCUCUUCUUUGAGUUCUA |
|  |  | A-065433-16 | GCAUCCACUGUGAAUGAUA |
| Mouse *DNMT3b* SMARTpool | E-044164-00-0010 | A-044164-09 | CUCUGAUAUUCUAAUGCCA |
|  |  | A-044164-10 | CCCUGAAACUUUAAAACUU |
|  |  | A-044164-11 | CCAUGAAAGUGAAUGACAA |
|  |  | A-044164-12 | UCAGGAUGAUAAAGAGUUU |
|  |  |  |  |
| Cy3-non-targeting siRNA #1 | D-001960-01-05 | only one siRNA | UGGUUUACAUGUCGACUAA |
| Cy3-*DNMT1* siRNA | GEHC4-000069 | only one siRNA | DY547-CCAUUGGCCUGGAGAUUAAUU |

**Table S2. Taqman probes used in the study**

| **Gene** | **Human probe** | **Mouse probe** |
| --- | --- | --- |
| *DNMT1* | Hs00945875_m1 | Mm01151063_m1 |
| *DNMT3a* | Hs01027166_m1 | Mm00432881_m1 |
| *DNMT3b* | Hs00171876_m1 | Mm01240113_m1 |
| *HLA-A* | Hs01058806_g1 |  |
| *HLA-B* | Hs00818803_g1 |  |
| *HLA-C* | Hs00740298_g1 |  |
| *H2-D1* |  | Mm00833934_g1 |
| *H2-K1* |  | Mm01612247_mH |
| *H2-D1/L* |  | Mm04208018_gH |
| *β2M* | Hs00984230_m1 | Mm00437762_m1 |
| *NNAT* |  | Mm00440480_m1 |
| *S100A10* |  | Mm00501458_g1 |
| *ACTB* | Hs01060665_g1 | Mm00607939_s1 |
| *GAPDH* | Hs99999905_m1 | Mm03302249_g1 |
| *Synaptophysin* |  | Mm00436850_m1 |
| *Rbfox3(NeuN)* |  | Mm01248771_m1 |
| *Calbinding* |  | Mm00486647_m1 |
| *Sl17a7 (VGlut1)* |  | Mm00812886_m1 |
